# Supplementary figures and images for: AIEdit: Alignment-free genome assembly polisher trained on spaced seed match patterns
Source: PLoS Comput Biol. 2026 May 6;22(5):e1014245. doi: 10.1371/journal.pcbi.1014245 (PMC13229362; doi:10.1371/journal.pcbi.1014245)

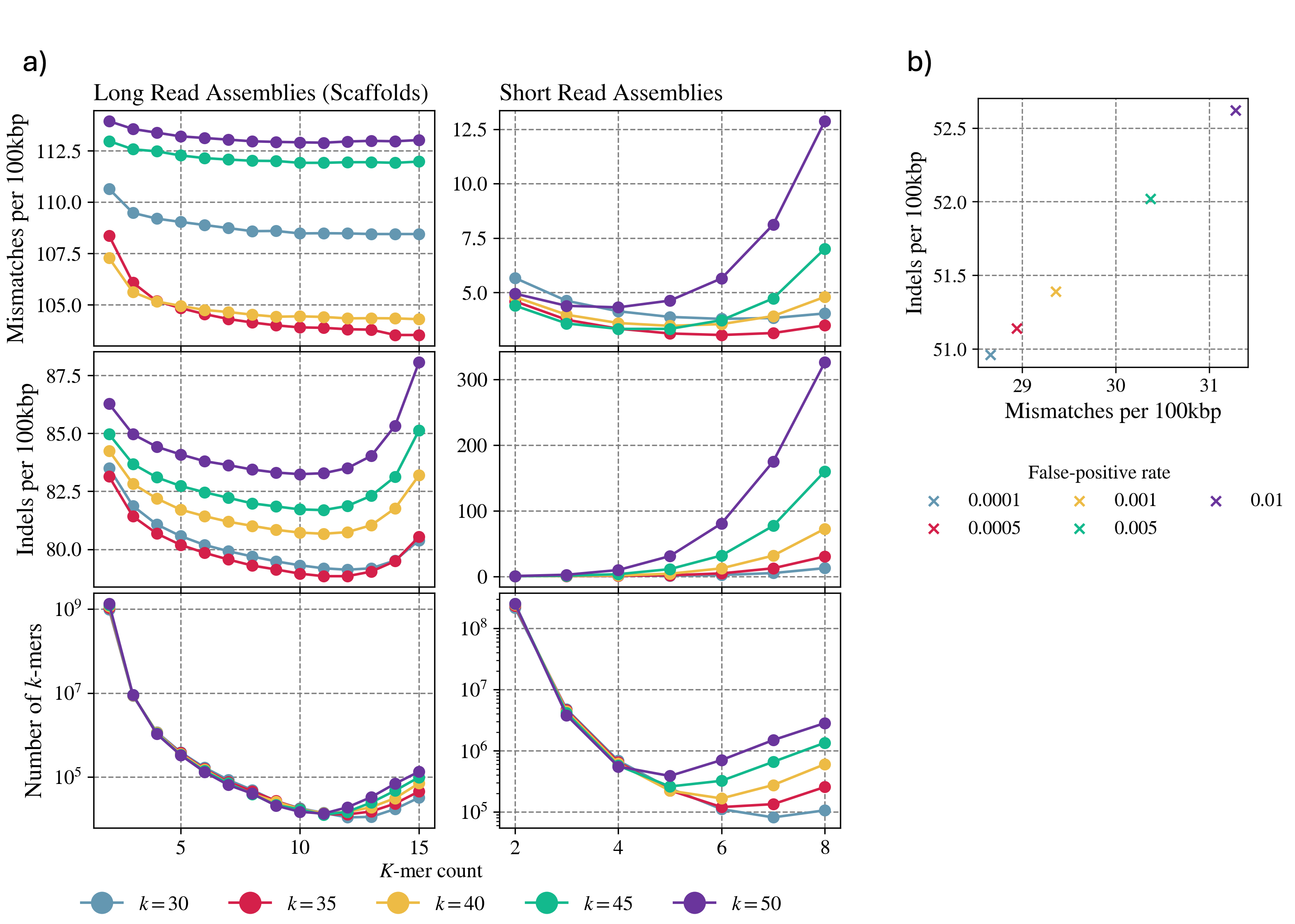

Supplement: S1 Fig — (a) AIEdit’s polishing accuracy across count thresholds in k-mer histograms, with colors representing different k-mer sizes. Short-read data and assemblies were simulated from the D. melanogaster reference genome using pIRS, and long-read datasets were generated using NanoSim and assembled with GoldRush. (b) Impact of Bloom filter false-positive rates (FPR) on error rates in the simulated long-read assembly. The plot demonstrates the model’s robustness to membership noise, showing that even a 100-fold increase in FPR results in a negligible increase in residual mismatches and indels (<3 per 100kbp). (PNG) [file pcbi.1014245.s001.png]

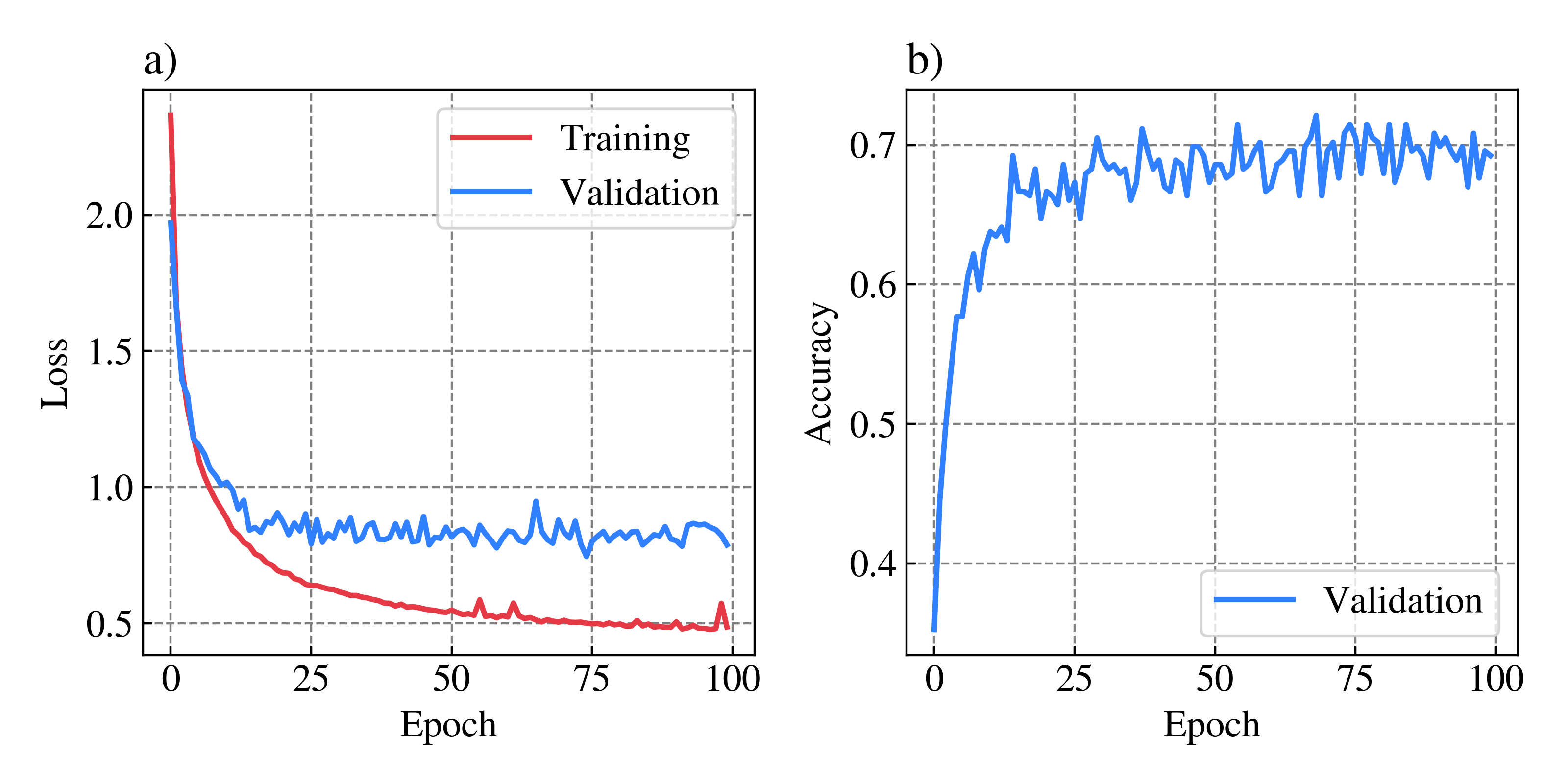

Supplement: S2 Fig — Training accuracy was not calculated to reduce training time. (PNG) [file pcbi.1014245.s002.png]

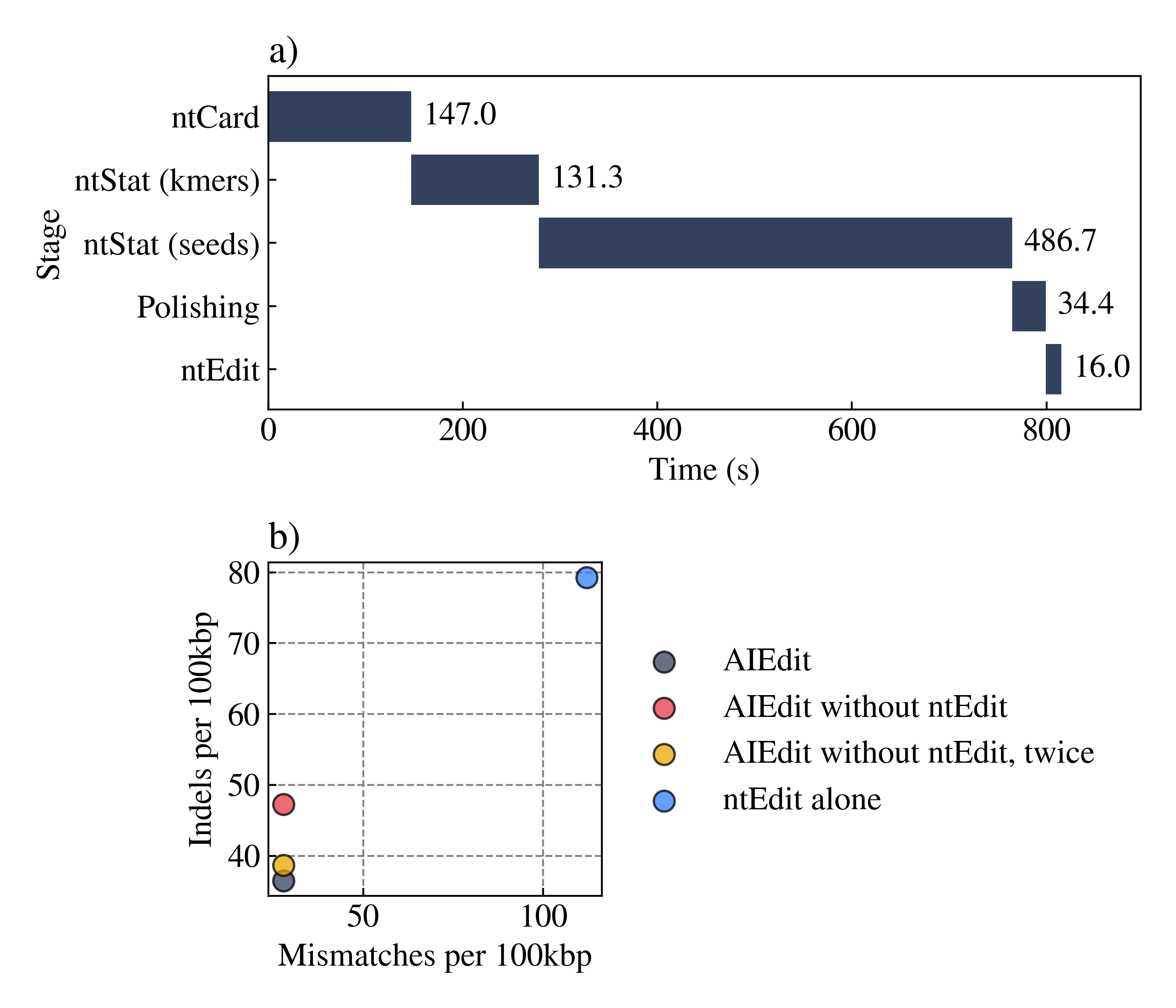

Supplement: S3 Fig — (PNG) [file pcbi.1014245.s003.png]

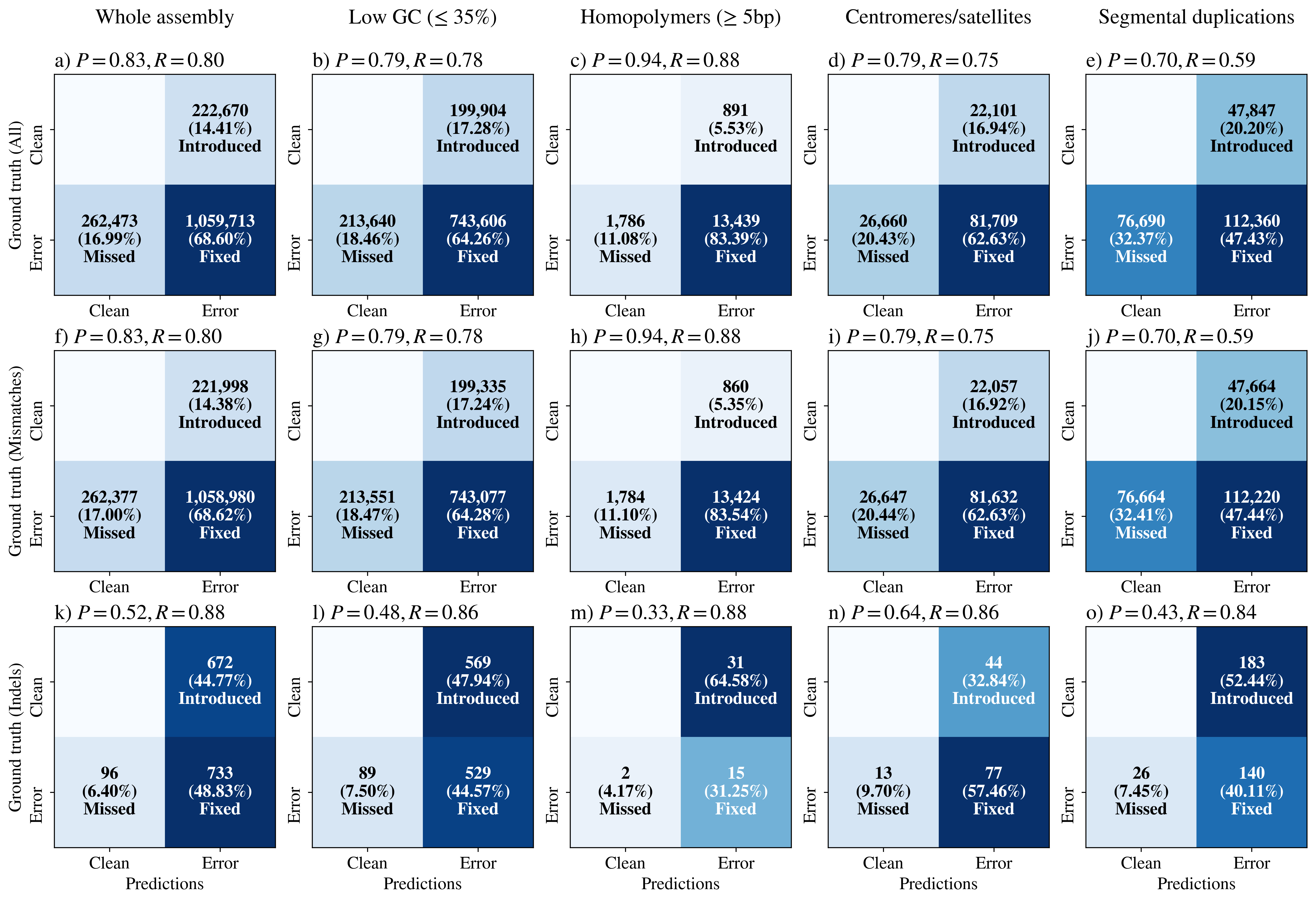

Supplement: S4 Fig — These results were gathered by aligning the draft and polished assemblies to the reference genome using minimap2, followed by variant calling with bcftools and intersection analysis using bcftools isec. Confusion matrices show the number of fixed, missed, and introduced errors across all errors (a-e), mismatches (f-j), and indels (k-o). Analysis is stratified by whole assembly, low GC content, homopolymers, centromeres/satellites, and segmental duplications. Genomic coordinates for repeats and segmental duplications were obtained from the UCSC Genome Browser and the marbl/CHM13 repository. The intensity of the blue colors are proportionate to the value in each cell normalized by the total number of edits in each confusion matrix (fixed + missed + introduced). (PNG) [file pcbi.1014245.s004.png]

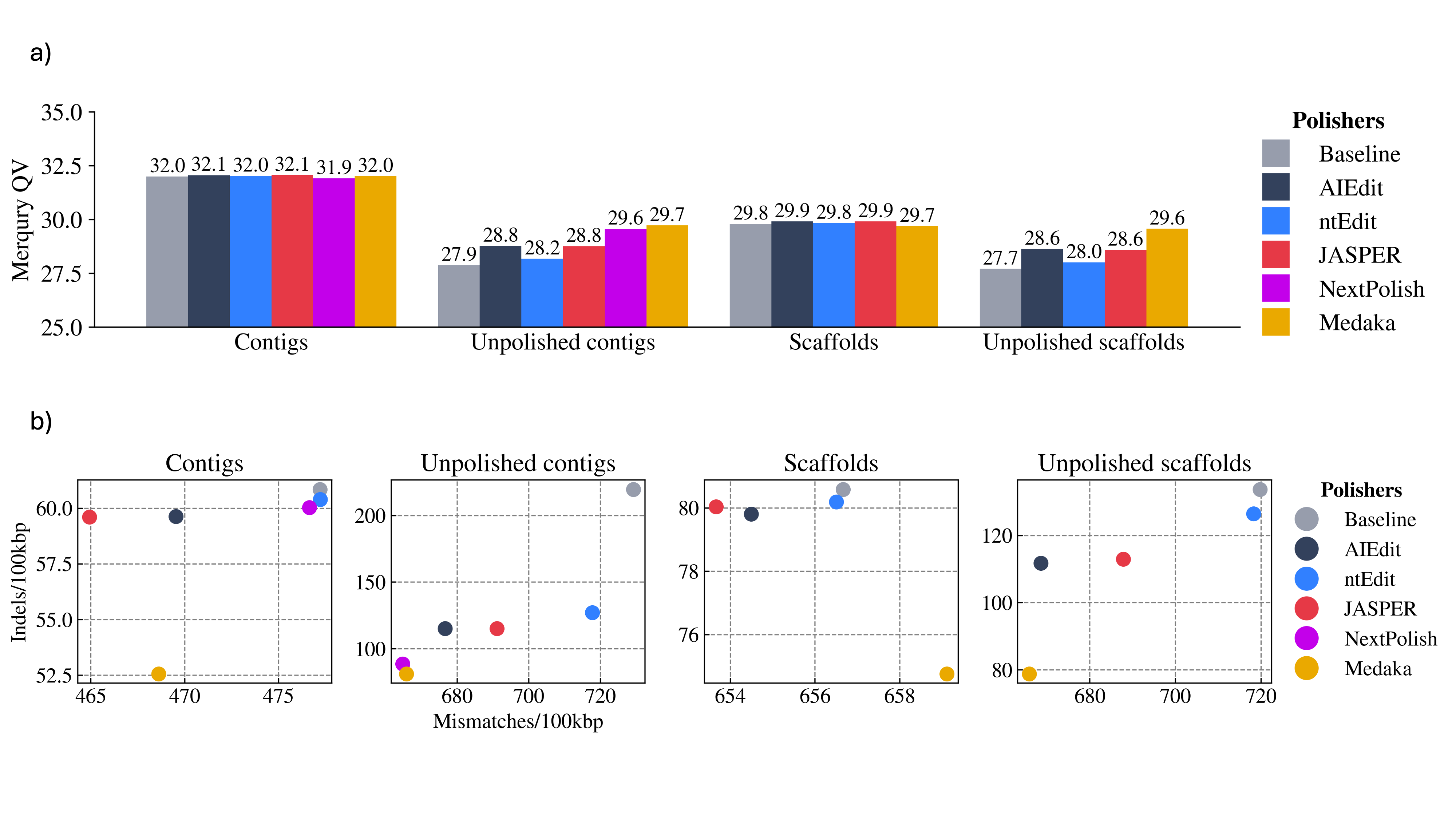

Supplement: S5 Fig — (a) Base-level accuracy as measured by Merqury for unpolished and polished contigs and scaffolds. AIEdit demonstrates a consistent improvement in QV scores, comparable to or exceeding established polishers. (b) Number of mismatches and indels per 100kbp. AIEdit effectively reduces both error types across all assembly stages, confirming its generalizability to different assembly algorithms and error profiles. (PNG) [file pcbi.1014245.s005.png]
