# Supplementary material for: AIEdit: Alignment-free genome assembly polisher trained on spaced seed match patterns
Source: PLoS Comput Biol. 2026 May 6;22(5):e1014245. doi: 10.1371/journal.pcbi.1014245 (PMC13229362; doi:10.1371/journal.pcbi.1014245)
Supplement: Attachment — Submitted filename: R2R.pdf [file pcbi.1014245.s012.pdf]

## Reviewer #1

This manuscript presents AIEdit, an alignment free assembly polishing pipeline that combines spaced seed membership signals from Bloom filters with a GRU based model to predict local edit patterns, followed by a final ntEdit round. The idea is promising, especially for long read assemblies where dense errors reduce the effectiveness of exact k-mer methods.

### Major revision requests

1. Evaluate further: For simulated data, report precision and recall of corrections separated into substitutions, insertions, deletions, and homopolymer contexts.

We appreciate the reviewer's request for a more granular evaluation of our polishing accuracy. To address this, we calculated the precision and recall of AIEdit's corrections for mismatches and indels across the draft assembly we generated from using simulated long reads derived from of the T2T human reference genome (Fig. 2f). These metrics were derived by aligning the draft and polished assemblies to the reference genome using minimap2, followed by variant calling with bcftools and intersection analysis using bcftools isec. As shown in Supplementary Figure S4, AIEdit maintains a high precision and recall (P=0.83, R=0.80), effectively balancing error correction with the avoidance of false positives in these simulated datasets. We have updated the results in Section 3.2 with these experiments.

2. Importance of AI components: Show AIEdit without the ML model (heuristic substitute) to show what the actual contribution is.

The neural network is the backbone of AIEdit. We now make the contributions of each algorithmic component clearer in the Discussion section. Without the error pattern detector, the pipeline would effectively function as a k-mer-based polisher utilizing spaced seeds for region identification. The core contribution of the ML model is its ability to detect mismatch signatures within a single k-mer using multiple spaced seeds. A heuristic approach is unsuitable here as the number of possible spaced seed hit/miss patterns increases exponentially with the k-mer size due to Bloom filter false-positives and the density of base errors within each k-mer, creating a search space too large for rule-based algorithms. As demonstrated in Supplementary Figure S3, the ML-based approach achieves significantly higher correction rates than ntEdit alone (which can act as a heuristic substitute), improving the Merqury QV from 31.0 to 32.9 on experimental ONT data by identifying dense error patterns that sparse k-mer heuristics typically miss.

3. Quantify robustness to Bloom filter false positives and parameter sensitivity: Training assumes ideal membership without Bloom filter false positives, but inference does not. Sweep Bloom filter false positive rate and show impact. Now it reads like dataset specific tuning.

We appreciate the reviewer's request to quantify the sensitivity of AIEdit to Bloom filter false-positive rates (FPR). We performed a parameter sweep across two orders of magnitude (FPR = 0.0001 to 0.01) to evaluate the impact on polishing accuracy and observed that while a higher FPR leads to a marginal increase in residual errors, the overall impact remains minimal. We observed that a 100-fold increase in FPR results in an increase of only 2.7 mismatches and 1.7 indels per 100kbp for the simulated *D. melanogaster* long read assembly. These results confirm that the model is inherently robust to the noise introduced by the Bloom filter. We have added this sensitivity analysis to Supplementary Fig. S1b and continue to utilize the defaults established in ntStat.

4. Stratify performance in difficult genomic contexts: Show error reduction stratified by repeats, low complexity, GC extremes, and homopolymer length.

We have now included a stratified analysis of error reduction, specifically targeting regions of low GC content (≤35%), homopolymers (≥5bp), centromeres/satellites, and segmental duplications (Supplementary Fig. S4). To perform this analysis, we generated custom BED files for GC extremes and homopolymer locations, while BED files for centromeres, satellites, and segmental duplications were obtained from the UCSC Genome Browser and the marbl/CHM13 GitHub repository. As illustrated, AIEdit remains robust in these difficult regions. Notably, in complex centromeric and satellite regions, it achieves a recall of 0.75, demonstrating the tool's effectiveness in preserving error-detection signals within repetitive landscapes.

5. Generalize beyond one assembler: Evaluate on at least one additional assembler output to show the method is not overly coupled to a specific assembly error profile. Clarify behavior on contigs vs scaffolds and gap handling. Also, what about metagenomics?

We appreciate the reviewer's feedback regarding the generalizability of AIEdit across different assemblers. To demonstrate that the method is not coupled to a specific assembler, we have included a new benchmarking analysis on assemblies generated by Flye, as shown in Supplementary Figure S5. AIEdit demonstrates consistent performance across both contig and scaffold levels, where it treats assembly gaps as potential error regions; if these gaps can be resolved as mismatches or small indels within the window size ($w$) supported by the model, AIEdit attempts to correct them. We have highlighted these points in the Methods and Results sections.

Regarding a possible application to metagenomics, our preliminary tests on Zymo R10 ONT data assembled with metaFlye (publicly available on the mikolmogorov/Flye GitHub repo) showed an error reduction from 1,133.73 mismatches and 899.54 indels per 100kbp to 1,099.00 and 844.13, respectively, using the combined reference of the 7 bacterial and 3 yeast genomes. To achieve this, we set the minimum count threshold to 2 to avoid filtering species-specific k-mers. While these initial results demonstrate that AIEdit can successfully process metagenomic datasets, we believe a comprehensive evaluation for metagenomic contexts requires specialized benchmarks and interpretation of k-mer interference across varying species abundances. Since the current study focuses on the AIEdit's algorithmic advancement and its performance on single-isolate genomes, we have opted to focus the manuscript on these high-confidence genomic results while confirming here that the tool is architecturally capable of handling metagenomic inputs.

**Minor points**

Clarify the operational definition of "alignment free" (especially relative to evaluation), and present reference-based vs reference free metrics with consistent interpretation.

We understand the potential ambiguity in our definitions of alignment-free and reference-based. In the context of AIEdit, alignment-free refers to the tool's operation, which relies on k-mers and spaced seed membership signals from Bloom filters, rather than requiring read-to-assembly alignments from tools like BWA or minimap2. While the polishing process itself and the Merqury quality assessments are performed *de novo*, we used QUAST as a reference-based metric. We have updated the Methods and Implementation sections to explicitly define these terms and ensure a consistent interpretation of how reference-free improvement (QV) differs from the reference-based error rates (mismatches and indels per 100kbp) reported in our results.

Overall, the method is interesting, but it needs a stronger experimental backbone and clearer attribution of improvements before the claims feel conclusive.

We thank the reviewer for their constructive feedback.

## Reviewer #2

The paper by Kazemi et al. describes AIEdit, a new alignment-free polisher for genome assemblies that uses ML. Polishing assemblies is an important problem in genome assembly pipelines to ensure accuracy in downstream genomics analyses, such as variant calling or genome annotation. AIEdit detects regions that need be corrected using low-abundance k-mers (contiguous and spaced) implemented as Bloom filters. Then, it uses a neural network (two GRUs

followed by a linear layer) to detect errors. AIEdit uses a spaced seed generator based on a genetic algorithm to maximize the support of possible error patterns. The manuscript is well-written and the content is interesting from a computational viewpoint.

**Major issues**

**(A) Parameters.**

(1) The choice of k appears to be good for AIEdit according to Suppl Figure 1, but what about the other tools? How was k chosen for the other tools?

We appreciate the reviewer’s comment regarding the selection of the k-mer sizes. To ensure a fair comparison, ntEdit was executed using the same k-mer size as AIEdit, which was set to 35 by default unless otherwise specified in the experiments. For JASPER, we utilized the tool’s own default setting of k=37, which we chose not to optimize as it is highly similar to our default value of 35 and represents a typical user experience of using the default parameters.

(2) The choice of the hyper-parameters for the neural network should to be discussed, e.g., layers, # of units, etc.

We appreciate the reviewer’s inquiry regarding the selection of hyperparameters for our neural network architecture. The choice of two GRUs followed by a linear layer was designed to capture both the structural information of spaced seeds and the local genomic context through the spaced seed hit/miss patterns, while maintaining a low computational footprint. In our empirical evaluations, we found that further increasing the number of layers or hidden units provided minimal gains in training and polishing quality. Therefore, we prioritized a simple architecture to ensure AIEdit remains a high-performance, alignment-free solution for large-scale assemblies. To support users with specialized needs, we have included various pretrained model configurations in our public repository. We have added this justification to the Implementation section of the manuscript.

(3) The choice of the parameter T and p needs to be discussed. The manuscript says "The parameters T and p can be specified by the user and are set to 3 and 0.5 by default." How are the results depends on the choice of these parameters? Under which circumstances users should change these parameters? Please discuss

We appreciate the reviewer’s inquiry regarding the sensitivity and selection of the *T* and *p* parameters. Parameter *T* controls the number of top-ranking error patterns evaluated for each region. Increasing *T* makes the polisher more robust as it examines more potential correction

patterns, though this comes with a slight increase in computational time. We observed that increasing *T* to 10 improves polishing performance in noisy nanopore datasets (Section 3.3). Parameter *p*, on the other hand, tunes the conservativeness of the tool by setting a minimum threshold for the expected improvement in k-mer confidence. Higher values of *p* ensure that AIEdit only applies edits when there is high certainty of improvement, which is particularly beneficial for reducing false-positive corrections in low-coverage datasets or highly heterozygous genomes. We have updated the Methods section to discuss these trade-offs and provide guidance on when users should consider adjusting these defaults to suit their specific genomic data.

**(B) Experiment results**

(1) In the manuscript AIEdit is compared against ntEdit, JASPER, POLCA and Medaka. NextPolish and Polypolish should be included for completeness. These two latter tools are considered good polishing tools (see, e.g., https://doi.org/10.1186/s12864-024-10582-x)

We thank the reviewer for the suggested comparators. Following this recommendation, we have included NextPolish in our benchmarking results (Figure 2), as it is a widely used tool for long-read assembly polishing. However, we did not include Polypolish in this study because it requires short-read sequencing data as input to polish assemblies from long reads. AIEdit is intended to operate using the same sequencing reads utilized for the initial assembly. We aimed to avoid the requirement for multiple sequencing technologies to ensure operational simplicity and broad accessibility for users, as many *de novo* projects aim for a single-technology solution to reduce the costs and complexities associated with preparing libraries for multiple sequencing platforms.

(2) Most polishing tools are known to introduce errors, not just correct them. What type of errors does AIEdit tend to introduce?

We appreciate the reviewer's observation that polishing tools can inadvertently introduce errors during the correction process. As shown in the stratified performance results we added to address Reviewer #1's request (Supplementary Fig. S4), AIEdit primarily introduces a small number of incorrect indels in regions characterized by large repeats and segmental duplications. This behavior is expected with k-mer-based polishers that utilize a relatively small context window, as the limited local signal in these highly repetitive landscapes can occasionally lead to incorrect error pattern predictions supported by many repetitive k-mers. We added text summarizing these observations in the Discussion section.

(2) The authors of some genome polishing tool recommend to use them repeatedly (2-3 rounds). Would running AIEdit more than once bring any benefit?

In our experiments, we found that the assembly quality does not change substantially after running the pipeline more than once with the same k-mer size. While it is theoretically possible to run the pipeline multiple times using a different k-mer size for each round to target varying error densities, the number of possible k-mer combinations would make the total runtime prohibitive for most large-scale applications. We have added this observation to the Discussion section to clarify that AIEdit is optimized to perform in a single pass.

(3) Can the author comment on the idea of using a pipeline of two different polishing tools to improve the quality of the assemblies even more?

We appreciate the reviewer's comment on the potential benefits of using multiple polishing tools in a pipeline. In fact, AIEdit is designed with this philosophy in mind. We integrate a round of ntEdit as a final post-processing step for the specific purpose of capturing any residual errors that the neural network might have missed. While a wide variety of polishing tools could theoretically be run in tandem to further improve assembly quality, each additional tool significantly increases the total runtime and computational overhead of the pipeline. We chose to integrate ntEdit specifically because its Bloom filter implementations are fully compatible with AIEdit, allowing for a seamless integration that adds only marginal overhead while effectively improving the final assembly quality. We clarified this point in the Discussion section.
